# Mitochondrial Genetic Diseases and Ophthalmic Manifestations: Molecular Pathophysiology, Genetics, and Clinical Management

**DOI:** 10.3390/ijms27146128

**Published:** 2026-07-09

**Authors:** Khaled K. Abu-Amero

**Affiliations:** Research Department, King Khaled Eye Specialist Hospital and Research Center, Riyadh 11462, Saudi Arabia; kamero@kkesh.med.sa; Tel.: +966-118401153

**Keywords:** mitochondria, oxidative stress, ophthalmology, diseases, genetics, clinical management

## Abstract

Mitochondrial genetic disorders compromise oxidative phosphorylation (OXPHOS) and cellular energy supply, and the eye is among the first organs to feel the deficit. Photoreceptors and retinal ganglion cells (RGCs) sustain among the highest metabolic rates in the body, so ophthalmic features often dominate the clinical picture and arrive before systemic disease is recognized. More than half of all patients with confirmed mitochondrial disease develop sight-threatening complications. This review integrates mtDNA and nuclear genetics; ophthalmic and extraocular phenotypes; the bioenergetic and apoptotic mechanisms that drive vision loss; the clinical examination and investigations that delineate the problem; the differential diagnoses that must be excluded; the contribution of common mtDNA haplogroup variation to age-related retinal disease; and the diagnostic, therapeutic, and counseling approaches that turn a molecular result into useful care. Recurring themes are heteroplasmy, the threshold effect, and the selective vulnerability of RGCs and extraocular muscle across genetically distinct disorders. Treatment remains largely supportive, but idebenone, gene therapy, mitophagy modulation, and targeted antioxidants now offer mechanism-based intervention for several ophthalmic manifestations.

## 1. Introduction

Mitochondrial genetic diseases are characterized by defects in oxidative phosphorylation (OXPHOS) and cellular energy production, and the eye is one of the first organs to show the deficit. The photoreceptors and retinal ganglion cells (RGCs) have among the highest metabolic rates in the body, and ocular findings are often the most conspicuous clinical findings prior to the diagnosis of systemic disease [1]. More than fifty percent of patients with mitochondrial disease have vision-threatening consequences. This review incorporates mitochondrial DNA and nuclear genetics; ophthalmic and extraocular phenotypes; bioenergetic and apoptotic mechanisms of vision loss; clinical assessments and investigations that clarify the issue; differential diagnoses to consider; effects of common mitochondrial DNA haplogroup variation on age-related retinal disease; and diagnostic, therapeutic, and counseling strategies to translate molecular findings into effective care. Heteroplasmy, the threshold effect and selective susceptibility of retinal ganglion cells and extraocular muscles in different genetic illnesses are recurring themes. Supportive care remains the mainstay of treatment, but mechanism-based interventions for various ocular symptoms are increasingly available via idebenone, gene therapy, mitophagy regulation, and targeted antioxidants.

## 2. Why the Eye Reports Mitochondrial Disease First

Mitochondria are double-membraned organelles whose five OXPHOS complexes generate adenosine triphosphate from electron flow coupled to proton pumping. The 16,569 bp circular mtDNA encodes 13 OXPHOS subunits, 22 transfer RNAs, and 2 ribosomal RNAs; the rest of the mitochondrial proteome (over 1500 proteins) is nuclear. Two routes, therefore, lead to mitochondrial disease: pathogenic variants in mtDNA and pathogenic variants in nuclear genes that build, maintain, or fuel the organelle. Both routes converge on the same downstream consequences: reduced ATP, increased reactive oxygen species (ROS), disrupted calcium handling, mitochondrial membrane potential collapse, and ultimately apoptosis. Nuclear gene mutations account for roughly 75% of mitochondrial disease cases, while mtDNA variants account for the remainder, a distinction that drives inheritance pattern, recurrence risk, and counseling strategy [2,3].

**Figure 1:**
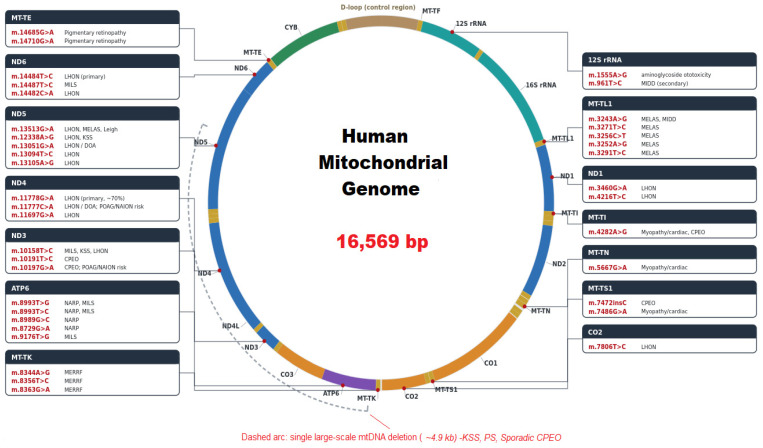
Pathogenic mtDNA point mutations mapped to the human mitochondrial genome.

Figure 1 maps the pathogenic mtDNA point mutations discussed in this review onto the 16,569 bp reference genome, with each variant tagged to its associated disease. Pathogenic point mutations in the human mitochondrial genome (rCRS, NC_012920, 16,569 bp), each tagged with the associated disease. The dashed arc marks the common single large-scale mtDNA deletion (~4.9 kb) associated with KSS, Pearson syndrome, and sporadic CPEO.

The retina is uniquely exposed. It consumes more oxygen per gram than any other tissue. More than 60% of retinal mitochondria sit in photoreceptor inner segments, where they fuel phototransduction and supply metabolic intermediates to the retinal pigment epithelium (RPE) and choroidal vasculature. In RGCs, mitochondria are distributed across the cell body, dendritic synapses, branch points, and unmyelinated axonal varicosities inside the globe. This polarized, energy-demanding architecture, combined with continuous light exposure and a polyunsaturated lipid environment, makes the retina an early-warning system: when OXPHOS falters, photoreceptors and RGCs fail before less demanding tissues. Common neuro-ophthalmic features therefore include bilateral optic neuropathy with disc pallor and visual field defects, chronic progressive external ophthalmoplegia (CPEO) with bilateral ptosis and limited motility, pigmentary retinopathy from salt-and-pepper mottling to frank retinal dystrophy, early cataracts, and retrochiasmal visual loss in syndromes with posterior pathway involvement [4,5].

Phenotypic heterogeneity, the hallmark of mitochondrial disease, has four sources. Heteroplasmy, the coexistence of mutant and wild-type mtDNA within a cell, sets the dose of the defect. The threshold effect means that biochemical and clinical disease appears only when mutant load exceeds a tissue-specific limit, and tissues with the highest ATP demand cross that threshold first. The mitochondrial bottleneck during oogenesis scatters heteroplasmy unpredictably between mother and child and between siblings, so siblings sharing a mother can range from clinically silent to severely affected. Nuclear background and environmental stressors including infection, exercise intensity, smoking, alcohol, and certain drugs modulate the final phenotype. Identical mtDNA variants therefore produce widely different presentations even within one pedigree, which is why mitochondrial disease is routinely missed on first clinical contact.

## 3. Primary Mitochondrial Optic Neuropathies

Mitochondrial dysfunction in the eye has a particular predilection for RGCs, whose axons converge to form the optic nerve. Mitochondria sit inside the RGC axons themselves, clustered at three critical sites: axonal varicosities, branch points, and synaptic terminals [6]. The unmyelinated intraocular portions of these axons depend entirely on locally distributed mitochondria for ATP production because axonal transport is too slow to supply distant energy demands, and these axons fire action potentials continuously. Small-caliber papillomacular fibers are particularly vulnerable because their high surface-to-volume ratio makes them metabolically expensive relative to their diameter, which is why Leber Hereditary Optic Neuropathy (LHON) damages this bundle preferentially. Once RGC axons cross the lamina cribrosa and enter the optic nerve proper, they become myelinated by oligodendrocytes and mitochondrial density drops sharply because myelin insulation reduces ATP demand. This anatomical transition explains why the LHON lesion is always anterior to the lamina, where unmyelinated energy-starved axons live and where OXPHOS collapse hits hardest [5]. The optic nerve therefore reveals mitochondrial disease at its source: the tightly packed, unmyelinated RGC axons inside the globe cannot tolerate the bioenergetic deficit that more sparsely innervated or myelinated tissues can survive. The two classical disease paradigms are LHON and autosomal dominant optic atrophy (ADOA, also written DOA). Both result from preferential loss of the small RGC axons within the papillomacular bundle that subserves central high-definition vision. Leber hereditary optic neuropathy is the commonest primary mitochondrial disease producing bilateral optic neuropathy and is the most frequent cause of isolated blindness in young men, with a point prevalence around 1 in 31,000. Onset is typically between 15 and 35 years, with subacute, painless, sequential loss of central vision, dense central scotomas, dyschromatopsia, microangiopathy, and disc swelling that resolves into optic atrophy. The second eye follows the first within weeks to a few months. Around half of patients experience some spontaneous recovery, but the proportion and depth of recovery depend strongly on the underlying mutation, and even when recovery occurs it is usually incomplete, limited to small fenestrations of visual field within the central scotoma. Three primary mtDNA point mutations affecting complex I subunits account for roughly 90% of cases: m.11778G>A in MT-ND4 (50–70% of families, the most prevalent worldwide, worst prognosis with roughly 4% recovery), m.14484T>C in MT-ND6 (10–15%, the most benign, best prognosis with 37–65% recovery, particularly if conversion occurs before age 20), and m.3460G>A in MT-ND1 (8–25%, the most severe biochemical phenotype with the lowest likelihood of spontaneous recovery). Mutations are typically homoplasmic, yet penetrance is incomplete and strongly male-biased. The lifetime risk of visual loss is about 50% for male carriers and about 10% for female carriers, which means LHON should be considered in any unexplained bilateral optic neuropathy regardless of sex [7]. This pattern points to oestrogen-mediated protection and modifier loci on the X chromosome. Around 10% of cases arise from rarer secondary mtDNA variants, so whole mitochondrial genome sequencing is warranted when the three primary mutations are negative and clinical suspicion is high [7]. The full set of pathogenic variants and their syndromic associations is given in Table 1 [8], and their positions on the mitochondrial genome are mapped in Figure 1. Mechanistically, complex I deficiency in LHON does two things simultaneously: it reduces ATP supply and increases premature electron leak from the respiratory chain, raising superoxide production [5]. Once a threshold is crossed that exceeds the cell’s compensatory capacity, an irreversible cascade precipitates apoptotic RGC death. RGCs of the papillomacular bundle are exquisitely sensitive because their unmyelinated intraocular axons are of small caliber and depend on local mitochondria for axonal transport and action potential propagation; RGC loss tracks tightly with axonal diameter. Two clinical correlates follow. First, RGC axonal swelling in the acute phase appears on OCT as peripapillary retinal nerve fiber layer thickening that proceeds in a specific order, starting temporally and inferiorly, then superiorly, and last nasally. Second, the pupillary light reflex is relatively preserved despite severe visual loss, because melanopsin-positive intrinsically photosensitive RGCs resist neurodegeneration. Two practical pitfalls deserve emphasis: in roughly 20% of acute LHON the fundus looks entirely normal and patients, especially children, are mislabeled as functional, and optic atrophy takes six to eight weeks to become clinically apparent. Triggers that precipitate conversion in genetically predisposed carriers include tobacco smoke, heavy alcohol consumption, antiretroviral therapy, head trauma, and severe infection, consistent with an environmental second hit. The evidence that smoking raises conversion risk is strong enough that carriers should be firmly advised not to smoke. Autosomal dominant optic atrophy (ADOA) is the most common inherited optic neuropathy and is caused predominantly by heterozygous loss-of-function variants in OPA1, a dynamin-family GTPase anchored to the inner mitochondrial membrane that regulates fusion, cristae organization, and apoptosis [9]. Between 50 and 70% of ADOA patients carry an OPA1 mutation [10]. Functional studies in patient fibroblasts show preserved basal ATP under glucose but a clear deficit in complex I-driven ATP synthesis under forced oxidative metabolism, with balloon-like reticular defects and complete fusion arrest in roughly half of cells. Different OPA1 mutations preferentially impair distinct functional domains: missense alleles V465F and V560F both block fusion and sensitize cells to apoptotic stimuli, but only V560F fails to restore membrane potential and limit cytochrome c release. The clinical phenotype is slow, symmetric, progressive central visual loss with temporal disc pallor and a centrocaecal scotoma, typically from the first decade and more indolent than LHON, though most patients are eventually registered legally blind. DOA-plus phenotypes add sensorineural hearing loss, peripheral neuropathy, ataxia, myopathy, and CPEO. A range of other nuclear genes, including *OPA3*, *ACO2*, *RTN4IP1* and *SSBP1*, cause optic atrophy with or without syndromic features (Table 1), broadening the differential beyond the two flagship neuropathies [8].

## 4. mtDNA Syndromes with Retinopathy, Ophthalmoplegia, and Multisystem Features

Mitochondria are unevenly distributed across the retina, concentrated where energy demand is highest. More than 60 percent sit in photoreceptor inner segments, where they fuel the constant ATP drain of phototransduction and supply metabolic intermediates to support the adjacent retinal pigment epithelium and choroidal blood supply. In retinal ganglion cells, mitochondria are scattered throughout the cell body, clustered at dendritic synapses, positioned at axonal branch points, and packed into unmyelinated axonal varicosities as they travel toward the optic nerve. This polarized, metabolically demanding architecture makes the retina uniquely vulnerable: it consumes more oxygen per gram than any other tissue in the body, so when OXPHOS falters, photoreceptors and RGCs fail before tissues with lower energy requirements even show stress [11]. The outer retina depends on mitochondrial ATP to maintain the light-capture machinery and supply the RPE, while the inner retina’s RGCs depend on mitochondria to sustain action potentials, axonal transport, and synaptic transmission. When a mitochondrial mutation or deletion reduces ATP production below the tissue-specific threshold, the retina is first to cross that line, which is why ophthalmic features often dominate the clinical picture and arrive before systemic disease is recognized. Single large-scale mtDNA deletions, ranging from 1.1 to 10 kb, cause CPEO, Kearns-Sayre syndrome (KSS), and Pearson syndrome. Larger deletions are generally associated with more severe disease. CPEO affects at least half of all patients with mitochondrial disease and presents with slowly progressive bilateral ptosis and symmetric ophthalmoparesis. Diplopia is usually absent because impairment is symmetrical, but about a third of patients develop symptomatic diplopia that needs active management. The extraocular muscles fail preferentially, and the degree of extraocular involvement frequently overshadows skeletal muscle weakness. This tissue-specific vulnerability has a basis: extraocular muscles are embryologically and genetically distinct from skeletal muscle, accumulate secondary mtDNA abnormalities such as multiple deletions far faster than skeletal muscle, and show a lower threshold before an OXPHOS defect becomes apparent. MRI consistently shows global extraocular muscle atrophy, and formal eye movement recordings indicate the ophthalmoplegia is mostly myopathic rather than supranuclear. KSS is a severe variant defined by an invariant triad: CPEO before age 20, pigmentary retinopathy, and cardiac conduction block, often with cerebellar ataxia, elevated CSF protein, sensorineural deafness, short stature, and endocrine dysfunction including diabetes, hypoparathyroidism, and growth hormone deficiency. Pearson syndrome presents in infancy with sideroblastic anaemia, pancytopaenia, and exocrine pancreatic failure; survivors often evolve a KSS phenotype in later childhood. Most large deletions are sporadic, but a small maternal transmission risk has been documented and warrants disclosure during counselling. The syndrome of sensory ataxic neuropathy with dysarthria and ophthalmoparesis (SANDO) sits alongside these, mostly in the context of mtDNA maintenance disorders secondary to POLG and PEO1 (C10orf2) mutations. When CPEO is associated with multiple mtDNA deletions or depletion rather than a single deletion, nuclear maintenance genes such as POLG, POLG2, C10orf2, RRM2B, TK2, and TYMP are implicated; the genetic spectrum is summarized in Table 1. Mitochondrial encephalomyopathy with lactic acidosis and stroke-like episodes (MELAS) is most often caused by the heteroplasmic m.3243A>G variant in MT-TL1. Stroke-like episodes typically begin before age 40 and do not respect vascular territories, producing cortical blindness, homonymous hemianopia, visual field defects, ptosis, and external ophthalmoplegia. Additional ocular features include cataracts, optic atrophy, and a characteristic pattern macular dystrophy. Systemic features comprise recurrent seizures, progressive dementia, sensorineural deafness, cardiomyopathy, and diabetes. Myoclonic epilepsy with ragged-red fibres (MERRF), usually caused by m.8344A>G in MT-TK, adds myoclonus, generalized seizures, ataxia, lipomatosis, and optic atrophy with progressive myopia and nystagmus. Neuropathy, ataxia, and retinitis pigmentosa (NARP) and maternally inherited Leigh syndrome both arise from MT-ATP6 m.8993T>G, with heteroplasmy level dictating phenotype: intermediate loads produce adult-onset NARP, loads above 90% produce infantile Leigh syndrome with rapid neurodegeneration. Maternally inherited diabetes and deafness (MIDD), also driven by m.3243A>G, adds macular pattern dystrophy to non-insulin-dependent diabetes and sensorineural hearing loss. Each syndrome shows that the same variant can give very different disease depending on heteroplasmy, tissue distribution, and modifier background. The RPE and photoreceptors of the outer retina can also be affected, producing either a generalized pigmentary retinopathy or a focal maculopathy. Pigmentary retinopathy may occur in isolation but more often appears as part of an extensive phenotype such as KSS, NARP, or maternally inherited Leigh syndrome. Less frequently, especially in children, early-onset cataracts develop that may need surgery depending on their effect on visual function.

## 5. Ophthalmological Manifestations in Primary Mitochondrial Disease

Clinical examination reveals a spectrum of findings depending on disease and severity. In a retrospective cohort of 74 patients with genetically confirmed primary mitochondrial disease, 35% had one or more ophthalmological abnormalities, with findings varying by syndrome. Retinal pigmentary changes were the most common at 16%, ranging from salt-and-pepper retinopathy to severe bone spicule formation and choriocapillaris atrophy. Partial or total optic atrophy occurred in 10%, decreased extraocular movement in 8%, with macular involvement, visual field defects, and surgically correctable findings such as strabismus and ptosis in smaller proportions [12]. Specific syndromes show characteristic patterns. CPEO patients universally presented with ptosis, while Pearson/Kearns-Sayre patients had high rates of both ptosis (66%) and retinal involvement (66%). MELAS patients with the m.3243A>G mutation showed retinal pigmentary changes in 25% of those examined in detail, within expected prevalence [13]. The diversity of findings underscores the need for standardized ophthalmological evaluation and long-term follow-up in all suspected mitochondrial disease. Any patient with unexplained retinal pigmentary changes, ocular motility defects, or ptosis warrants screening for underlying mitochondrial disease [12].

## 6. Clinical Examination and Investigations

The clinician carries the burden of delineating the ocular problem while also looking for, and excluding, more disseminated systemic disease and treatable mimics. Visual acuity is measured with a Snellen or LogMAR chart, and a formal refraction is sometimes needed to confirm that subnormal vision is not simply a refractive error, particularly in children. The examination is then focused on the relevant site of pathology. Ptosis should be assessed directly, since compensatory frontalis overaction can mask it; a rough grade of mild, moderate, or severe suffices in a general setting, but surgical candidates need the margin reflex distance, levator function, and frontalis power measured. Most CPEO patients show orbicularis oculi weakness, which is a useful pointer. The range of eye movements and any saccadic slowing should be documented, along with the symmetry between eyes and the pattern of any strabismus. Patients with symptomatic diplopia should see an experienced orthoptist to judge whether corrective prisms would help. Pupillary light reflexes and any relative afferent pupillary defect must be checked before dilating drops are instilled; preservation of the pupillary reflex despite dense central scotoma is characteristic of mitochondrial optic neuropathy. A slit lamp examination of the anterior and posterior segments completes the assessment, and a dilated fundus examination of the macula and peripheral retina is essential because pigmentary changes are easily missed [14]. Optical coherence tomography gives high-resolution structural assessment of the optic nerve head and individual retinal layers, and in LHON it captures the early peripapillary nerve fiber layer swelling and the segmental thinning that follows. Fluorescein angiography looks for optic nerve leakage, macular oedema, and vasculitis; the absence of disc leakage in acute LHON is a useful diagnostic clue that separates it from inflammatory optic neuropathy. A baseline fundus photograph is recommended for future comparison, and fundus autofluorescence shows the true extent of RPE dysfunction and whether the fovea is involved. Formal perimetry, static or kinetic, defines the field defect, although the dense central or centrocaecal scotoma of LHON makes accurate perimetry difficult. Visual electrophysiology to ISCEV standards separates primary RGC disease from outer retinal dysfunction: full-field ERG assesses photoreceptor and inner retinal function, the P50 and N95 components of the pattern ERG are sensitive to RGC damage and can detect early conversion in LHON, and pattern and flash visual evoked potentials show delayed latency and reduced amplitude in optic neuropathy, becoming undetectable in severe long-standing disease. Contrast-enhanced MRI of the brain and orbit is mandatory in any unexplained optic neuropathy to exclude compressive, infiltrative, and demyelinating causes; atypical chiasmal or optic nerve enhancement has been reported in LHON, and orbital MRI in CPEO shows the characteristic global extraocular muscle atrophy. The systemic workup is conducted with a neurologist or internist and includes screening for cardiac conduction defects with an electrocardiogram, given the lethal potential of block in KSS.

## 7. Differential Diagnosis

Because treatment delay can cost reversible vision in the mimics, the differential diagnosis is not academic. It is the central clinical task in any first presentation of unexplained optic neuropathy or progressive ophthalmoplegia.

### 7.1. LHON Mimics

Demyelinating optic neuritis is the leading alternative in a young adult. It is usually unilateral, worsens over about two weeks, and is accompanied in roughly 90% of cases by pain on eye movement. There is a relative afferent pupillary defect with marked dyschromatopsia, the disc is normal in most cases because the lesion is retrobulbar, and visual recovery begins within four to six weeks with an excellent prognosis. MRI white matter lesions predict progression to multiple sclerosis. Several red flags should redirect the workup away from typical demyelination: bilateral simultaneous or rapidly sequential involvement with poor recovery, marked disc swelling with hemorrhages or exudates, and systemic autoimmune features. Neuromyelitis optica should be considered specifically, with aquaporin-4 and myelin oligodendrocyte glycoprotein antibody testing and spinal cord MRI. Toxic and nutritional optic neuropathy presents at any age with bilateral, slowly progressive, painless loss, color vision affected early and out of proportion to acuity, and a caecocentral scotoma that overlaps with LHON and ADOA; a careful history exposes alcohol and tobacco use, poor diet, malabsorption, and mitochondrial-toxic drugs such as ethambutol and linezolid. Compressive and infiltrative causes, including optic nerve glioma in children, meningioma in middle age, lymphoma, and metastases, must be excluded with contrast-enhanced MRI of the brain and orbit using fat-suppression sequences, particularly when the course is indolent as in ADOA.

### 7.2. CPEO Mimics

Age-related dehiscence of the levator aponeurosis is the commonest cause of bilateral ptosis over age 50, especially in contact lens wearers, and the surgical history in this review’s source material includes a patient nearly operated on for involutional ptosis before CPEO was recognized on the day of surgery. Three diseases must be actively excluded. Myasthenia gravis overlaps closely because it favors the extraocular muscles and the orbicularis oculi, but it is distinguished by variability, fatigability, diurnal worsening, and the absence of the fixed, symmetrical pattern of CPEO; acetylcholine receptor antibodies are negative in 40 to 60% of purely ocular cases, single-fiber EMG of the orbicularis is sensitive but specialist-dependent, and a response to acetylcholinesterase inhibitor or steroids supports the diagnosis. Oculopharyngeal muscular dystrophy, an autosomal dominant PABPN1 trinucleotide repeat disorder more common in French-Canadian and Ashkenazi Jewish populations, has a later onset from the fifth decade, presents with ptosis followed by dysphagia and proximal weakness, and rarely causes complete ophthalmoplegia or diplopia; every suspected CPEO patient should be asked specifically about swallowing and choking. Congenital cranial dysinnervation disorders [15], including congenital fibrosis of the extraocular muscles, present in early childhood with non-progressive ophthalmoplegia, worse vertical than horizontal gaze, and a compensatory head posture; the early onset and non-progressive course distinguish them from classical CPEO [16]. The presence of pigmentary retinopathy alongside ophthalmoplegia should always prompt consideration of KSS and its cardiac risk [17].

## 8. Mechanisms of Vision Loss: ROS, Quality Control, and RGC Apoptosis

Across LHON, ADOA, KSS, AMD, and glaucoma [18,19], vision loss converges on three interlocking mechanisms. First, OXPHOS dysfunction increases premature electron release from complexes I and III, generating superoxide and downstream ROS that overwhelm retinal antioxidant defenses already taxed by light and lipid peroxidation. Superoxide dismutase (SOD) activity and glutathione levels become depleted, shifting the redox balance toward persistent oxidative stress [20,21]. Mitochondrial normal function is compromised when demand exceeds the capacity. Impaired mitochondria are not removed by mitophagy quickly enough to keep up with the damage, and the mitochondrial unfolded protein response (mtUPR) does not restore balance to protein levels. Mitophagy encompasses three principal pathways: PINK1/Parkin-mediated ubiquitination of impaired mitochondria (the predominant pathway in most cell types), BNIP3-induced hypoxic responses, and FUNDC1-mediated membrane receptor interaction with autophagy machinery. When these pathways malfunction, accumulated damaged mitochondrial proteins and lipids exacerbate dysfunction. In experimental models, concurrent activation of mitophagy and mtUPR rescues cell viability, restores membrane potential, and maintains mitochondrial mass under oxidative stress. Mitochondrial biogenesis, governed by PGC-1 alpha and TFAM, fails to keep pace, and fission-fusion balance shifts towards fission with fragmented networks unable to support distal axonal compartments. In glaucoma, retinal ganglion cells die when mitophagy fails. OPTN mutations block the clearance of damaged mitochondria, triggering oxidative damage and ferroptosis. This connects a specific genetic defect directly to disease mechanisms. AMD works differently. The retinal pigment epithelium can’t clear its mitochondria efficiently. You see low PGC-1 alpha, high lipocalin-2, and autophagy grinds to a halt. The RPE deteriorates as a result. Diabetic retinopathy creates a different problem. High glucose floods cells with reactive oxygen species that mitochondria can’t handle on their own. But here’s the key: if PINK1 and Parkin signaling stays functional, cells compensate. Mitophagy ramps up and restores balance. Additionally, sustained bioenergetic insufficiency and ROS trigger intrinsic apoptosis in RGCs through cytochrome c release, caspase activation, and neurotrophic factor withdrawal. RGC apoptosis is the proximate cause of irreversible visual field loss in glaucoma [22] and the final common pathway in LHON and ADOA [23]. Why RGCs in particular? Their continuous high ATP demand for sustained action potential firing, polarized morphology requiring distributed axonal mitochondria, dependence on axonal transport along unmyelinated intraocular axons, and sensitivity to ROS-driven apoptosis combine to make them the first cell type to cross the bioenergetic threshold. This shared vulnerability explains why diverse genetic and physiological insults, from primary mtDNA mutation in LHON to elevated intraocular pressure in glaucoma, produce a fundus picture that converges on disc pallor and a centrocaecal or arcuate field defect. It also explains why mechanism-based therapy aimed at restoring bioenergetics or limiting oxidative damage has rational appeal across genetically distinct disorders [24].

## 9. Oxidative Stress and Age-Related Ocular Diseases

Mitochondrial dysfunction is not confined to monogenic syndromes. Diabetic retinopathy, glaucoma, AMD, and cataracts all involve mitochondrial oxidative stress as a major driver. AMD, the leading cause of irreversible central vision loss in older adults, is driven by oxidative damage to the RPE, which is exposed daily to a constant lipid-oxidative load from phagocytosing photoreceptor outer segments. Impaired mitochondrial function in the RPE produces declining autophagic capacity, lipofuscin accumulation in the form of A2E, drusen formation, and chronic inflammation that culminates in photoreceptor loss. mtDNA is particularly susceptible to ROS-induced mutation and deletion because of its proximity to the inner membrane, its lack of protective histones, and its more limited repair machinery; the burden of mtDNA damage correlates positively with AMD progression while repair capacity correlates negatively [25]. Common mtDNA haplogroup variation further modifies risk: haplogroup J roughly doubles AMD risk, haplogroup H is not significant, and the combined JTU cluster is found in 34% of AMD patients versus 15% of controls (odds ratio roughly 2.99) [26]. These population-level associations operate independently of nuclear risk variants and likely reflect haplogroup-specific differences in OXPHOS coupling efficiency, ROS generation, mitochondrial calcium handling, and stress response thresholds shaped by ancient population-level selection in different climates [27,28]. Similar patterns are seen in glaucoma. Increased IOP causes mechanical and metabolic stress of RGC axons at the lamina cribrosa. In genetically predisposed individuals, mitochondrial dysfunction and oxidative stress accelerate RGC apoptosis and visual field loss. Specific mtDNA susceptibility variants and haplogroup distributions have been described in primary open-angle glaucoma [27] and non-arteritic anterior ischemic optic neuropathy [29], with overrepresentation of haplogroups T and U in the cases. Glaucoma patients showed 20% less mitochondrial respiratory function and more mtDNA mutations in clinical studies. In experimental models, the mitochondrion-targeted antioxidant SkQ1 attenuated glaucomatous changes, suggesting that targeting mitochondria is a strategy beyond IOP reduction alone [30]. Diabetic retinopathy shows analogous haplogroup associations, implicating mitochondrial function in vascular endothelial damage from chronic hyperglycaemia. Chronic hyperglycemia triggers oxidative stress through polyol pathway flux, advanced glycation end products (AGEs), and protein kinase C (PKC) activation, increasing ROS production beyond retinal antioxidant capacity. Similar haplogroup associations are also found in diabetic retinopathy, implicating mitochondrial function in vascular endothelial damage by chronic hyperglycemia. Chronic hyperglycemia causes oxidative stress via polyol pathway flux, activation of advanced glycation end products (AGEs), and protein kinase C (PKC) and increases ROS production beyond the antioxidant capacity of the retina [31]. Mitochondria are both the main source of ROS and a major target of oxidative damage, thereby forming a vicious cycle. High glucose inhibits mitochondrial antioxidant enzymes (MnSOD, GSH); enhances mtDNA damage; enhances membrane permeability; and induces mitochondrial swelling, leading to cytochrome c release and retinal capillary cell apoptosis [32].

### Cataract Formation and Mitochondrial Oxidative Stress

Mitochondria are abundant in lens epithelial and differentiating fiber cells but absent from mature lens fibers, accounting for approximately 90% of total oxygen consumption in the lens. Lens epithelial cells are metabolically active and maintain fiber cell function via gap junctions, providing ATP and regulating intracellular calcium. The lens exists in a naturally hypoxic environment [33]. Lipid peroxidation (LPO) is a major mechanism of cataract genesis. Primary products include diene conjugates and lipid hydroperoxides, while end products are fluorescent substances that accumulate during aging. Aqueous humor from cataract patients shows 24 to 42-fold higher fluorescent lipid peroxidation products compared to normal lenses. Phospholipid hydroperoxides escape enzymatic detoxification and trigger chain propagation of LPO, causing membrane damage, altered permeability, and protein aggregation [34]. In fact, lenses generate superoxide and hydrogen peroxide as normal physiological byproducts. The generation rates for these are inversely proportional to the life time of the lens. Cataracts are correlated with maturity. Mitochondrial complexes I and III are the primary producers of reactive oxygen species (ROS). Healthy lenses contain antioxidant enzymes (superoxide dismutase, catalase and glutathione peroxidase) and chemical scavengers (including glutathione and ascorbate). In mature cataracts, the activity of glutathione peroxidase is decreased, levels of glutathione are lower and the function of superoxide dismutase (SOD) is impaired, suggesting a depletion of antioxidant reserves [35]. Production of free radicals from lens reductants is catalyzed by transition metal ions such as copper and iron. Copper concentrations increase with age and are higher in diabetes. The reduction of hydrogen peroxide, catalyzed by these metals, results in the formation of hydroxyl radicals that induce lipid peroxidation (LPO). EDTA chelation almost completely prevents lipid peroxidation, which supports the role of metal ions in this process. This paradox is the reason why high concentrations of antioxidants do not prevent cataracts. Reductants can become pro-oxidants in the presence of catalytically active metal ions [36].

## 10. Molecular Mechanisms in Inherited Age-Related Eye Disorders

Inherited mutations that affect mitochondrial function, in combination with age related disease, lead to early onset retinal degeneration via accumulation of oxidative damage. Respiratory chain dysfunction and impaired energy production in energy-dependent tissues, like the optic nerve and retina, are caused by mutations in inherited mitochondrial DNA and nuclear genes that disrupt mitochondrial DNA replication and repair. Inherited diseases are caused by primary mtDNA mutations (LHON, MELAS) and mutations in nuclear genes involved in mtDNA replication and repair (e.g., DNA polymerase gamma, Twinkle helicase, adenine nucleotide translocator). The severity of the disease is determined by heteroplasmy and clinical features often manifest at 80–90% mutant mtDNA levels. Age-related diseases are caused by stochastic damage to mitochondrial DNA that accumulates over decades. Mitochondrial DNA is more susceptible to damage than nuclear DNA because of its close proximity to the inner membrane where reactive oxygen species are generated and lack of nucleotide excision repair. Oxidative damage generates mutagenic lesions and mitochondrial DNA repair capacity decreases with aging. The persistent overproduction of reactive oxygen species impairs ATP synthesis, cellular survival pathways and promotes apoptosis. Main pathogenic factors are mtDNA point mutations and deletions in respiratory complexes, defective mtDNA replication and repair, nucleotide pool imbalances that increase mutation rates, reactive oxygen species from mitochondrial and non-mitochondrial sources (e.g., NADPH oxidase and photosensitizers) and concurrent damage to mitochondrial proteins, lipids, and DNA. When the load of mtDNA mutations exceeds a certain threshold, mitochondrial bioenergetic failure will cause an increase in ROS production and the resulting mutagenesis. Therapeutic intervention is complicated by the appearance of metabolic memory, the persistence of oxidative damage long after the initial factors such as hyperglycemia in diabetes have been removed. Therapeutic strategies to prevent progression of disease should target mtDNA replication, repair, nucleotide precursor availability or reactive oxygen species production.

**Figure 2:**
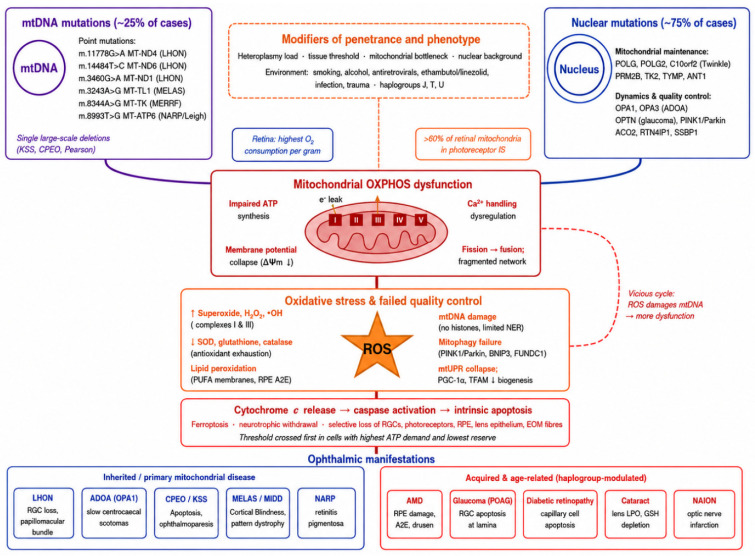
The genetic origin of mitochondrial oxidative stress and convergence on ophthalmic disease.

Figure 2 shows the genetic origin of mitochondrial oxidative stress and convergence on ophthalmic disease. mtDNA and nuclear mutations converge on OXPHOS failure, ROS accumulation and selective loss of high-demand ocular cells.

## 11. Exosome-Mediated Intercellular Communication in Retinal and Optic Nerve Mitochondrial Impairment

Recent research identifies exosomes and other small extracellular vesicles as active mediators of mitochondrial communication between cells, rather than mere passive remnants. This redefines mitochondrial failure in retinal and optic nerve tissue as a widespread problem, rather than one confined to the originating cell. Cells consistently encapsulate mtDNA and mitochondrial proteins into extracellular vesicles through a selective sorting mechanism: quality-control pathways redirect oxidized and damaged mitochondrial components away from vesicles under normal conditions; however, this gating mechanism is impaired under stress [37]. Upon release, mtDNA acts as a damage-associated molecular pattern. It attaches to innate immune receptors and initiates inflammatory signaling in recipient cells [38]. Mitochondria-derived vesicles containing these mitochondrial DAMPs can activate microglia and trigger pro-inflammatory cytokine release, thereby linking mitochondrial damage to neuroinflammation. In the retina and optic nerve, where microglial activation and oxidative stress are recognized features of ganglion cell degeneration, this pathway offers a plausible mechanism for the dissemination of localized mitochondrial dysfunction into extensive tissue inflammation. Alongside the encapsulated mtDNA in exosomes, regulatory microRNA may also be encapsulated within exosomes. In conditions of oxidative stress, retinal pigment epithelial cells selectively package specific miRNAs into exosomes for secretion, which subsequently regulate reactive oxygen species levels, apoptosis, and the production of inflammatory cytokines in recipient retinal cells [39]. The direction of effect is inconsistent. Exosomal miRNAs can either exacerbate or alleviate damage depending on the state of the parent cell and the cargo, which is why the same vesicular biology is being assessed as both a pathogenic factor and a therapeutic agent. Exosomes are a novel cell-free approach for corneal regeneration. Corneal epithelial, stromal and endothelial cell-derived exosomes have been shown to promote epithelial wound closure and enhance proliferation and migration by activating HSP27, STAT and beta-catenin signaling [40]. In addition, exosomes produced by mesenchymal stem cells derived from iPSCs enhance these benefits by promoting epithelial and stromal regeneration and decreasing scar formation. This phenomenon is attributed to miR-432-5p that suppresses collagen deposition through TRAM2 [41]. It must be appreciated that both lines of evidence are preclinical and thus their clinical significance remains to be established.

## 12. Clinical Management of Mitochondrial Eye Disease

Treatment is largely supportive, and management spans the primary ocular pathology, related systemic disease, and rehabilitation. Patients with visual impairment should be seen at least once in a low vision aids clinic, and the coordinating ophthalmologist should facilitate registration with local social services. Where eligible, formal registration as legally blind or severely sight-impaired gives access to additional support, including financial benefits. Children need care coordinated with community pediatrics and education services to keep them in mainstream schooling where possible. Many affected patients are young adults who were previously well, and the psychological impact is heavy; occupational rehabilitation and liaison with employers often allows them to keep working in an adapted role. To stop secondary diseases from worsening the primary pathology, patients should be screened for systemic conditions, particularly diabetes mellitus, and these should be actively managed.

### 12.1. LHON

There is no proven prophylaxis for at-risk carriers and no intervention that reliably prevents involvement of the second eye after conversion. Management is largely supportive, with visual and occupational rehabilitation and regular follow-up to document progression and any spontaneous recovery. Idebenone, a short-chain ubiquinone analog that crosses the blood–brain barrier and improves mitochondrial ATP synthesis with postulated antioxidant properties, is the only approved agent. A multicenter double-blind randomized controlled trial and subsequent studies support a consistent visual benefit in a proportion of treated patients, best when started early in the acute phase. Idebenone does not reverse established optic nerve damage, but responders show an increased rate and likelihood of recovery compared with natural history. EPI-743, an alpha-tocotrienol-quinone with antioxidant properties, and gene therapy by allotopic expression of MT-ND4 delivered intravitreally with a modified adeno-associated virus vector are under investigation but remain confined to clinical trials, and clear proof of efficacy has not yet been established. Carriers should be counselled to avoid smoking and heavy drinking.

### 12.2. CPEO, Diplopia, and Ptosis

About a third of CPEO patients develop diplopia, usually worse at near because of impaired convergence. Angles of deviation should be measured at near and distance, and the directions of maximal diplopia documented with formal orthoptic assessment. Most cases are managed with corrective prisms incorporated into the patient’s glasses once a stable strength is reached. Extraocular muscle surgery is rarely done because the disease is progressive and strabismus recurs, but botulinum toxin or maximal horizontal recti surgery can be offered for a large cosmetically troubling deviation after appropriate counseling about aims and limitations [42]. Ptosis surgery can substantially improve quality of life but demands a conservative approach by an experienced oculoplastic surgeon. Overcorrection in CPEO risks sight-threatening corneal exposure, magnified by orbicularis weakness, dry eye, and a poor Bell phenomenon [43]. The intervention is tailored to residual levator function, frontalis power, and cosmetic preference. The two common procedures are anterior levator resection to maximize upward muscle action and, where levator function is insufficient, a brow suspension using a silicone sling or autologous fascia lata to harness the frontalis. Lid height can drop again because the disease is progressive, and revision is not always advisable when the risk of exposure keratopathy is high. Dry eye is common from blepharitis and poor tear film wetting; daily lid hygiene and preservative-free lubricants reduce the risk of corneal epithelial toxicity. A confirmed CPEO diagnosis has implications for relatives, so appropriate genetic counseling, including reproductive advice where relevant, should be offered [43,44].

### 12.3. Diabetic Retinopathy, Glaucoma, and AMD

The treatment of diabetic retinopathy involves restoring mitochondrial function by using SOD mimetics like MnTBAP, which inhibit superoxide production, suppress cytochrome c translocation, and block caspase-3 activation. An adjunctive benefit is provided by metabolic agents that support ATP production and antioxidant defense. Mitophagy enhancers, antioxidants, and bioenergetic support are promising additions to IOP reduction strategies that are neuroprotective and target mitochondrial dysfunction in glaucoma. Nicotinamide supplementation in a Phase II glaucoma trial showed benefit by enhancing mitochondrial quality control. In AMD, antioxidants are confirmed in the AREDS study to slow progression, with real potential for mitophagy modulation. Preclinical evidence for melatonin, trehalose, and notoginsenoside R1 as mitophagy activators Real obstacles hamper clinical translation: most drugs are blocked by the blood-retinal barrier, there are no reliable biomarkers for mitophagic flux in patients, and rodent models do not reliably predict human outcomes. Inter-patient variability in AMD and diabetic retinopathy: the case for patient stratification in future trials [45].

## 13. Diagnosis, Therapy, and Genetic Counselling

Diagnosis begins with clinical phenotyping and a careful maternal pedigree, because maternal-only transmission alongside ophthalmic plus systemic features is the strongest pointer to a mitochondrial etiology. Reduced penetrance frequently masks the maternal pattern, so the absence of other affected relatives does not exclude LHON. Initial workup includes visual acuity, color vision, automated visual fields, fundus photography, optical coherence tomography of the macula and retinal nerve fiber layer, electroretinography where retinal involvement is suspected, audiometry, electrocardiography with Holter monitoring, lactate measurement, and brain MRI if neurological features are present. Next-generation sequencing of the entire mitochondrial genome with deep coverage detects point mutations and quantifies heteroplasmy with sensitivity far above Sanger sequencing. Long-range PCR or Southern blot identifies single large-scale deletions in muscle. Comprehensive mtDNA testing should generally precede nuclear panels because mtDNA variants account for roughly a quarter of cases, and a positive result immediately guides counseling. When mtDNA testing is negative, nuclear gene panels or whole-exome sequencing is appropriate; an expanding catalogue of more than 350 nuclear genes now causes mitochondrial disease [46]. Muscle biopsy with COX/SDH histochemistry and respiratory chain enzyme assays retains a role in ambiguous cases or when atypical features require exclusion of other myopathies, and the characteristic findings are a mosaic of COX-negative fibers and ragged-red fibers [47]. POLG deserves particular attention: it encodes the only DNA polymerase responsible for mtDNA replication, and recessive mutations cause infantile-onset mtDNA depletion syndromes with hepatic failure and PEO, while dominant mutations cause adult-onset CPEO with myopathy [48]. Therapy is mechanism-based rather than curative [36]. Idebenone is the only agent approved specifically for mitochondrial disease. The RHODOS, RHODOS-OFU, and LEROS trials in LHON showed the best outcomes when treatment is started early, within 12 months of onset; continued for at least 24 months at 900 mg/day in three divided doses; and is most effective in m.11778G>A and chronic m.14484T>C eyes. Coenzyme Q10 and ubiquinol provide complementary antioxidant support at 200 to 500 mg daily. Gene therapy uses allotopic expression: a nuclear-encoded copy of the relevant mitochondrial gene is delivered by adeno-associated virus into the RGC nucleus, the polypeptide is synthesized in the cytoplasm, and it is imported into mitochondria via an N-terminal targeting sequence. The RESCUE trial [49] and REVERSE [50] phase III trials of lenadogene nolparvovec in 11778G>A LHON missed their primary endpoint of treated-versus-sham visual acuity at 48 weeks. Both trails shows that there are no difference in the degree of improvement was observed compared with sham-treated eyes. A new therapeutic avenue targets autophagy and mitophagy, the cellular quality-control pathways that clear damaged organelles. In both OHT and MYOC-mutant glaucoma models, chronically elevated IOP produces an accumulation of morphologically abnormal mitochondria in RGC somas and optic nerve axons, accompanied by oxidative DNA damage (8-OHdG elevation). Mechanistically, mitophagy flux declines before RGC loss occurs, implying that defective mitochondrial removal drives neurodegeneration rather than the reverse. p62 and LC3B accumulate, and autophagic flux to lysosomes is suppressed, yet lysosomes themselves remain intact. RGC-specific Atg5 knockout (which blocks autophagy) recapitulates the phenotype in the absence of IOP elevation: damaged mitochondria accumulate, oxidative stress rises, and the mice lose approximately 39% of RGCs and 59% of axons within six weeks. This demonstrates that basal autophagy is essential for RGC survival. Pharmacological restoration of autophagy via the mTOR inhibitor Torin 2 raises mitophagy flux within 24 h. In OHT models, a single intravitreal dose of Torin 2 preserves RGC function (pattern ERG amplitudes 31.8 vs. 17.4 microvolts in controls), increases RGC counts by 37%, restores axonal transport to the superior colliculus, and clears abnormal mitochondria without changing IOP. In human retinal explants subject to neurotrophic deprivation, Torin 2 increases RGC survival by approximately 50%. These findings suggest that mTOR inhibition or other autophagy activators that bypass IOP-dependent pathways may offer neuroprotection in glaucoma, particularly in cases where pressure control alone is insufficient [51]. Arginine and citrulline relieve acute stroke-like episodes in MELAS via nitric oxide pathways; L-carnitine, B vitamins, and vitamin E provide adjunctive metabolic support. Mitochondrial transplantation and base editing of mtDNA are at preclinical and early clinical stages and may eventually allow direct correction. Across approaches, early intervention is critical because RGC loss is irreversible and the therapeutic window closes quickly [52]. Genetic counseling is difficult because mtDNA does not follow Mendelian rules. Patients fall into three counseling groups. Those with a nuclear gene mutation are the most straightforward: risk follows standard autosomal or X-linked rules, and prenatal diagnosis by direct mutation testing is reliable. Those with a respiratory chain enzyme defect but no identified variant is the hardest: the underlying gene may be nuclear or mitochondrial, recurrence risk is empirical, and counseling depends on pedigree analysis. Those with an identified mtDNA variant lie between, with transmission predictable in direction but not in dose because of the bottleneck and threshold effect. Roughly a quarter of mtDNA point mutations are de novo. A homoplasmic carrier transmits the mutation to all children, but penetrance still varies. A heteroplasmic carrier transmits a variable load, so siblings range from asymptomatic to severely affected. Single large-scale deletions are usually sporadic with low recurrence risk [53]. Reproductive options include oocyte donation, prenatal diagnosis by chorionic villus sampling or amniocentesis, preimplantation genetic testing, and mitochondrial replacement therapy by pronuclear or spindle transfer [53]. Prenatal and preimplantation testing are most informative at extreme heteroplasmy values; intermediate loads are difficult to interpret because embryonic and adult tissue heteroplasmy does not correspond cleanly. Donor oocytes eliminate transmission risk for mtDNA variants entirely but are not acceptable to all families. Mitochondrial replacement therapy is available only in selected jurisdictions and remains under regulatory and ethical review. LHON illustrates the difficulty plainly: the three primary mutations are typically homoplasmic with incomplete, male-biased penetrance, so prenatal and preimplantation testing are uninformative, and donor oocytes are the only reliable way to prevent transmission. OPA1-related ADOA, being autosomal dominant, is amenable to standard prenatal testing. Heteroplasmic syndromes such as MELAS and NARP/MILS sit between these poles, where prenatal testing can stratify embryos by mutant load but cannot eliminate residual uncertainty. Counseling extends beyond reproductive planning to family screening, psychological support, and connection with disease-specific patient organizations; population-based studies confirm that early access to specialist counseling improves diagnostic yield and reproductive decision-making [44,54].

Mitochondrial gene editing targets a distinct issue: the direct correction of pathogenic mtDNA variants. The primary instrument is the DddA-derived cytosine base editor (DdCBE), which effectuates precise modifications in mtDNA without inducing a double-strand break [55]. AAV-mediated DdCBE has demonstrated efficacy in vivo in mouse post-mitotic tissue [56], and a compensatory edit partially ameliorated a pathogenic mt-tRNA mutation in mice. The aforementioned study serves as a warning: the maximum vector dose resulted in significant off-target editing and serious adverse effects, indicating a narrow therapeutic window [57]. No mitochondrial base editing has commenced clinical trials.

## 14. Drug Delivery Strategies for Mitochondrial Therapy

Mitochondrial therapy will only work if the drugs can actually reach the damaged mitochondria at a high enough concentration. Three strategies to target drugs to mitochondria are discussed: (I) taking advantage of the high negative membrane potential of the inner mitochondrial membrane to deliver lipophilic cationic compounds, (II) using mitochondrial enzymes to catalyze the release of drugs from prodrugs, and (III) transporter-mediated delivery of prodrugs. Mitochondria-targeted antioxidants with lipophilic triphenylphosphonium (TPP) conjugates are accumulated several hundredfold in mitochondria due to the electrochemical gradient. Mitochondrion-targeted CoQ10 analogues and SOD mimetics that inhibit superoxide production, suppress cytochrome c translocation, and block caspase-3 activation [58]. N-acetylcarnosine, an ophthalmic prodrug, and mitochondrial antioxidants address ROS scavenging and lipid membrane repair. L-carnosine is a hydroxyl radical scavenger. Combinatorial approaches targeting several points in the oxidative damage cascade have shown promise in preclinical models. clinical trials [59]. Acetyl-L-carnitine, omega-3 fatty acids, and coenzyme Q10 improved visual function and stabilized early AMD.

## 15. Utilization of Stem Cells as a Novel Approach for Mitochondrial Therapy

Stem cell biology has generated two areas of research pertinent to retinal ganglion cell and optic nerve disorders. Induced pluripotent stem cells (iPSCs) can differentiate into retinal ganglion cells and neural progenitors, and their derivatives safeguard injured neurons in animal models. In a rat optic nerve crush model, intravitreal iPSC-derived extracellular vesicles enhanced ganglion cell survival, maintained nerve fiber layer thickness, and facilitated axonal regeneration via PI3K/AKT signaling [60]. Human pluripotent stem cell-derived neural progenitors provided equivalent protection in a rat optic nerve compression model, enhancing neurotrophic factor expression and reducing inflammatory markers in the retina and optic nerve [61]. Mesenchymal stem cells (MSCs) primarily function via paracrine signaling rather than through cell replacement, thereby prioritizing neuroprotection. This method has impacted patients. A phase II trial administered allogeneic bone marrow-derived mesenchymal stem cells intravitreally to five patients with acute non-arteritic ischemic optic neuropathy. The cells were well tolerated intraocularly, and four patients exhibited visual improvement; however, one developed an epiretinal membrane, and the small uncontrolled design restricts any efficacy claims [62]. The outcome advocates for more extensive randomized trials rather than for clinical implementation.

## 16. Conclusions

Mitochondrial disease produces a recognizable ophthalmic signature: optic neuropathy, ophthalmoplegia, pigmentary retinopathy, and accelerated age-related retinal degeneration because the retina sits at the top of the body’s metabolic demand curve. The underlying genetics span mtDNA point mutations, large deletions, nuclear genes encoding OXPHOS components and maintenance machinery, and haplogroup-level variation that modifies risk for common disease. The clinical work is to recognize mitochondrial disease early, to exclude the treatable mimics that share its presentation, to use the narrow therapeutic window for idebenone and gene therapy in patients who fit the evidence, and to give families counseling that respects the limits of mtDNA prediction. Progress in mitophagy modulation, base editing of mtDNA, mitochondrial transplantation, and targeted antioxidants is real but not yet curative, and the most consequential decisions remain diagnostic, counseling, and rehabilitative decisions made early in the disease course. Ophthalmologists, geneticists, and mitochondrial disease specialists working together can change disease trajectory and preserve sight, even where the underlying defect cannot yet be cured.

## Figures and Tables

**Table 1:** Mitochondrial syndromes and ophthalmic diseases associated with mtDNA mutations: genetic variants and clinical features.

Syndrome	Acronym	Genetic Variant	Clinical Features
Leber Hereditary Optic Neuropathy	LHON	Primary mtDNA mutations: MT-ND4 (m.11778G>A, ~70%); MT-ND1 (m.3460G>A, ~15%); MT-ND6 (m.14484T>C, ~10%). Additional variants: m.10158T>C, m.11697G>A, m.13513G>A, m.12338A>G, m.13094T>C, m.13105A>G, m.13051G>A, m.4216T>C, m.7806T>C, m.11777C>A. Other complex I genes: MT-ND2, MT-ND3, MT-ND4L, MT-ND5; also MT-ATP6, MT-CO3, MT-CYB. Nuclear: NDUFS2, NDUFAF5, DNAJC30, MCAT. Maternal inheritance with incomplete penetrance; males more severely affected.	Acute to subacute, painless, bilateral sequential visual failure due to optic neuropathy; central scotomas; red-green dyschromatopsia; optic atrophy. Variable expressivity. LHON-plus features may include myopathy, ataxia, peripheral neuropathy, movement disorder, multiple sclerosis-like disease, cardiac abnormalities, endocrine dysfunction, hearing loss, and kidney disease.
Dominant Optic Atrophy	DOA	Nuclear: OPA1 (most common). Maternally inherited isolated optic atrophy variants: MT-ND4 (m.11777C>A), MT-ND6 (m.13051G>A), MT-ATP6 (m.8993T>G, heteroplasmy-dependent).	Slow, progressive, relatively symmetric bilateral visual failure due to optic neuropathy; optic atrophy with temporal disc pallor; visual field defects; childhood to adulthood onset. DOA-plus may include myopathy, ataxia, peripheral neuropathy, sensorineural hearing loss.
Chronic Progressive External Ophthalmoplegia	CPEO	Sporadic CPEO: single large de novo mtDNA deletions (2.3–9.5 kb); larger deletions cause more severe disease. Point mutations: m.10197G>A, m.10191T>C, m.11777C>A, m.7472insC, m.7490_7497delAAAAGAA, m.13513G>A, m.4282A>G. Inherited CPEO: autosomal dominant POLG1, POLG2, ANT1, C10orf2 (twinkle), RRM2B, DNA2, OPA1; autosomal recessive TYMP, POLG1, DGUOK, TK2, MGM1, RNASEH1. Maternal: MT-TL1, MT-TQ, MT-TA, MT-TY, MT-TK, MT-TN, MT-TI, MT-TP.	Progressive bilateral ptosis; diffuse ophthalmoplegia with limited eye movements; weakened eyelid closure; secondary myopia; pigmentary retinopathy in some cases. CPEO-plus may feature ataxia, parkinsonism, seizures, tremor, peripheral neuropathy, gastrointestinal dysmotility, proximal myopathy, cardiomyopathy, hearing loss, hypogonadism.
Kearns-Sayre Syndrome	KSS	Single large mtDNA deletions (typically 1.2–8 kb; commonly 4.9–7.4 kb, 30–90% heteroplasmy). Associated point mutations: m.12338A>G, m.10158T>C, m.14453G>A. mtDNA duplications also identified.	Onset before age 20 years with CPEO; salt-and-pepper pigmentary retinopathy; ptosis; external ophthalmoplegia; optic nerve hypoplasia; night blindness. CSF protein > 1 g/L; cerebellar ataxia; cardiac conduction block; sensorineural hearing loss; myopathy; dysphagia; diabetes; renal tubular dysfunction; hypoparathyroidism; endocrine dysfunction.
Pearson Syndrome	PS	Single large mtDNA deletions (1.3–9.5 kb). mtDNA duplications described.	Infantile sideroblastic anaemia; pancytopaenia; exocrine pancreatic failure; renal tubular defects; failure to thrive. Survivors may evolve into KSS/CPEO phenotype with ptosis, ophthalmoplegia, pigmentary retinopathy.
Neuropathy, Ataxia, Retinitis Pigmentosa	NARP	MT-ATP6: m.8993T>G (heteroplasmy > 70%), m.8993T>C (rarely), m.8989G>C, m.8729G>A. Heteroplasmy level inversely correlates with severity.	Late-childhood or adult-onset axonal sensorimotor peripheral neuropathy; cerebellar ataxia; pigmentary retinopathy progressing to retinitis pigmentosa with progressive night blindness, visual field constriction, photopsia, eventual central vision loss; basal ganglia lucencies; seizures; developmental delay (infantile onset with higher heteroplasmy).
Maternally Inherited Leigh Syndrome	MILS	MT-ATP6 m.8993T>G with heteroplasmy > 90%; MT-ND3 (m.10158T>C, m.9030C>A), MT-ND5, MT-ND6 (m.14487T>C), MT-ND2 (m.6930C>T), MT-ND4 (m.9176T>G).	Infantile onset (3–12 months); subacute relapsing encephalopathy; progressive psychomotor regression; cerebellar and brainstem signs; raised lactate in blood or CSF; bilateral white matter and basal ganglia lesions on neuroimaging; pigmentary retinopathy; optic atrophy; nystagmus; ophthalmoplegia; visual impairment; seizures; hypotonia; respiratory failure. Rapid progression and high mortality.
Maternally Inherited Diabetes and Deafness	MIDD	MT-TL1 (m.3243A>G). Secondary 12S rRNA variants (m.1555A>G, m.961T>C). Exclusively maternal transmission; high penetrance in heteroplasmic carriers.	Non-insulin-dependent diabetes mellitus; bilateral sensorineural deafness; macular pattern dystrophy; short stature; cardiac abnormalities; myopathy; proteinuria; gastrointestinal disease. Variable ophthalmic involvement includes cataracts, retinopathy, progressive myopia, ptosis.
Mitochondrial Encephalomyopathy, Lactic Acidosis and Stroke-Like Episodes	MELAS	MT-TL1 (m.3243A>G, ~80%, heteroplasmy 50–90%). Secondary: m.3256C>T, m.3291T>C, m.3271T>C, m.3252A>G, m.13513G>A.	Stroke-like episodes at age < 40 years (cortical blindness, homonymous hemianopia, visual field defects); seizures and/or dementia; ragged-red fibres and/or lactic acidosis; ptosis; external ophthalmoplegia; pigmentary retinopathy (variable); optic atrophy; cataracts; diabetes mellitus; cardiomyopathy; bilateral sensorineural deafness; cerebellar ataxia; progressive neurodegeneration.
Myoclonic Epilepsy and Ragged Red Fibres	MERRF	MT-TK (m.8344A>G ~80%, m.8356T>C, m.8363G>A, m.8361G>A). Additional tRNA variants: MT-TF, MT-TH, MT-TI, MT-TL1, MT-TP, MT-TS1, MT-TS2. Other m.8296A>G, m.8289T>C.	Myoclonus; generalized seizures (progressive myoclonic epilepsy); cerebellar ataxia; myopathy with ragged-red fibres on biopsy; raised lactate in blood or CSF; dementia; myopathic ptosis; progressive external ophthalmoplegia; optic atrophy; pigmentary retinopathy; visual field constriction; bilateral sensorineural deafness; peripheral neuropathy; spasticity; multiple lipomata; cardiomyopathy.
Mitochondrial Neurogastrointestinal Encephalopathy	MNGIE	TYMP biallelic pathogenic variants (thymidine phosphorylase deficiency leading to mtDNA proliferation and secondary depletion); LIG3 biallelic pathogenic variants.	Severe gastrointestinal dysmotility; cachexia; ptosis; external ophthalmoplegia; sensorimotor neuropathy; diffusely abnormal white matter on neuroimaging; optic nerve atrophy.
Kjellin Syndrome	SPG15	ZFYVE26 biallelic pathogenic variants.	Hereditary spastic paraplegia with pigmentary maculopathy in some individuals; cognitive impairment.
ACO2-associated Disease	OPA9	ACO2 biallelic pathogenic variants.	Retinal degeneration; optic atrophy; variable neurological involvement.
RTN4IP1-associated Disease	OPA10	RTN4IP1 biallelic pathogenic variants.	Optic atrophy; mild retinal degeneration in some individuals.
SSBP1-associated Disease	OPA3	SSBP1 monoallelic and biallelic pathogenic variants.	Optic atrophy; variable degree of retinal dystrophy and foveopathy; sensorineural hearing loss in some individuals.
FDXR-associated Disease	—	FDXR biallelic pathogenic variants.	Variable pigmentary retinopathy; optic atrophy; auditory neuropathy.
Primary Coenzyme Q10 Deficiency	—	Biallelic defects in CoQ10 biosynthesis pathway genes (COQ2, COQ4, COQ6, COQ8A, COQ8B, COQ9, PDSS1, PDSS2).	Retinitis pigmentosa; encephalopathy; nephrotic syndrome; cerebellar ataxia; cardiomyopathy. Responsive to CoQ10 supplementation in some cases.
POLG-associated Disease	—	POLG (autosomal dominant or recessive).	Pigmentary retinopathy; PEO; ataxia-neuropathy spectrum; Alpers syndrome; sensorimotor neuropathy; hepatocerebral disease.
Pigmentary Retinopathy in Severe Neurological Phenotype	—	MT-TE variants (m.14685G>A, m.14710G>A); other tRNA variants affecting retinal energy metabolism.	Pigmentary retinopathy; ptosis; ophthalmoplegia; severe neurological involvement.
Maternally Inherited Sensorineural Hearing Loss with Optic Atrophy	—	Multiple mitochondrial tRNA mutations; 12S rRNA m.1555A>G associated with aminoglycoside-induced ototoxicity.	Progressive sensorineural hearing loss; bilateral optic nerve atrophy; visual field loss; variable multisystem involvement.
Mitochondrial Myopathy with Cardiac Involvement	—	m.4282A>G (MT-TI), m.5667G>A (MT-TN), m.7486G>A (MT-TS1).	Proximal myopathy; hypertrophic or dilated cardiomyopathy; conduction defects; ptosis; ophthalmoplegia.
Primary Open-Angle Glaucoma (mtDNA-associated)	POAG	Susceptibility variants: m.10158T>C (MT-ND3), m.11777C>A, m.13051G>A, m.10197G>A, m.3243A>G, m.14484T>C, m.8344A>G, m.15674T>C. Haplogroup H and J protective; T and U increase risk.	Elevated intraocular pressure; progressive optic nerve cupping; arcuate scotomas and other visual field defects; optic nerve pallor and atrophy. Mitochondrial dysfunction in retinal ganglion cells compromises ATP production, oxidative stress defense, and axonal transport. Chronic, often asymptomatic until advanced; maternal inheritance pattern when mtDNA-driven.
Non-Arteritic Anterior Ischemic Optic Neuropathy (mtDNA-associated)	NAION	Susceptibility variants: m.11777C>A, m.10197G>A, m.13051G>A, m.3243A>G, m.11778G>A, m.8993T>G. Haplogroups T and U increase risk through impaired oxidative phosphorylation.	Sudden, painless monocular vision loss; altitudinal (usually superior) visual field defect; optic disc hyperaemia followed by pallor; relative afferent pupillary defect; dyschromatopsia. Compromised microcirculation to the optic nerve head from mitochondrial insufficiency in vascular endothelium. Risk factors: small disc-to-cup ratio, diabetes, hypertension, elevated homocysteine. Acute onset distinguishes from LHON; no recovery of vision.

Abbreviations: mtDNA, mitochondrial DNA; CSF, cerebrospinal fluid; PEO, progressive external ophthalmoplegia. Genetic variants are listed by gene with representative pathogenic mtDNA point mutations (m. notation), large-scale deletions, and nuclear-encoded mitochondrial genes where relevant. Heteroplasmy levels modulate phenotypic severity in many maternally inherited syndromes.

## Data Availability

No new data were generated for this review. All cited studies are referenced and publicly available through their respective journals.

## Abbreviations

The following abbreviations are used in this manuscript:

A2E	N-retinyliden-N-retinylethanolamine
AAV	Adeno-associated virus
ACO2	Aconitase 2
ADOA	Autosomal dominant optic atrophy
AGEs	Advanced glycation end products
AMD	Age-related macular degeneration
AREDS	Age-Related Eye Disease Study
ATP	Adenosine triphosphate
BNIP3	BCL2-interacting protein 3
C10orf2	Chromosome 10 open reading frame 2 (Twinkle helicase)
CAC	Cytotoxic T-lymphocyte-associated protein
CNS	Central nervous system
COX	Cytochrome c oxidase
CPEO	Chronic progressive external ophthalmoplegia
CSF	Cerebrospinal fluid
CoQ10	Coenzyme Q10
DNA	Deoxyribonucleic acid
DOA	Dominant optic atrophy
EDTA	Ethylenediaminetetraacetic acid
EPI-743	EPI-743 (alpha-tocotrienol-quinone)
ERG	Electroretinogram
ETDRS	Early Treatment Diabetic Retinopathy Study
FDXR	Ferredoxin reductase
FUNDC1	FUN14 domain containing 1
GSH	Glutathione
IOP	Intraocular pressure
ISCEV	International Society for Clinical Electrophysiology of Vision
Idebenone	Idebenone (short-chain ubiquinone analog)
KSS	Kearns-Sayre syndrome
L-carnitine	L-carnitine
LHON	Leber hereditary optic neuropathy
LIG3	DNA ligase III
LPO	Lipid peroxidation
LogMAR	Logarithm of the minimum angle of resolution
MELAS	Mitochondrial encephalomyopathy with lactic acidosis and stroke-like episodes
MERRF	Myoclonic epilepsy with ragged-red fibres
MIDD	Maternally inherited diabetes and deafness
MILS	Maternally inherited Leigh syndrome
MRI	Magnetic resonance imaging
MT-ND1	Mitochondrial NADH dehydrogenase subunit 1
MT-ND4	Mitochondrial NADH dehydrogenase subunit 4
MT-ND6	Mitochondrial NADH dehydrogenase subunit 6
MT-TK	Mitochondrial tRNA for lysine
MT-TL1	Mitochondrial tRNA for leucine 1
MYOC	Myocilin
MnSOD	Manganese superoxide dismutase
MnTBAP	Manganese tetrakis
NAD	Nicotinamide adenine dinucleotide
NADPH	Nicotinamide adenine dinucleotide phosphate
NAION	Non-arteritic anterior ischemic optic neuropathy
NARP	Neuropathy, ataxia, and retinitis pigmentosa
NGS	Next-generation sequencing
OCT	Optical coherence tomography
OHT	Ocular hypertension
OPA1	Optic atrophy 1
OPA3	Optic atrophy 3
OPTN	Optineurin
OXPHOS	Oxidative phosphorylation
PCR	Polymerase chain reaction
PEO	Progressive external ophthalmoplegia
PEO1	Progressive external ophthalmoplegia 1
PGC-1 alpha	Peroxisome proliferator-activated receptor gamma coactivator 1-alpha
PINK1	PTEN-induced kinase 1
PKC	Protein kinase C
POAG	Primary open-angle glaucoma
POLG	DNA polymerase gamma
POLG2	DNA polymerase gamma 2
Parkin	Parkin
RGCs	Retinal ganglion cells
RNA	Ribonucleic acid
ROS	Reactive oxygen species
RPE	Retinal pigment epithelium
RRM2B	Ribonucleotide reductase M2B
RTN4IP1	Reticulon 4-interacting protein 1
SANDO	Syndrome of sensory ataxic neuropathy with dysarthria and ophthalmoparesis
SDH	Succinate dehydrogenase
SOD	Superoxide dismutase
SSBP1	Single-strand DNA-binding protein 1
SkQ1	SkQ1 (mitochondrion-targeted antioxidant)
TFAM	Transcription factor A, mitochondrial
TK2	Thymidine kinase 2
TPP	Triphenylphosphonium
TYMP	Thymidine phosphorylase
mTOR	Mechanistic target of rapamycin
mtDNA	Mitochondrial DNA
mtUPR	Mitochondrial unfolded protein response
ubiquinol	Ubiquinol (reduced coenzyme Q10)
